# Gene Expression Profiling of Lacrimal Glands Identifies the Ectopic Expression of MHC II on Glandular Cells as a Presymptomatic Feature in a Mouse Model of Primary Sjögren's Syndrome

**DOI:** 10.3389/fimmu.2018.02362

**Published:** 2018-10-31

**Authors:** Junping Yin, Junfeng Zheng, Fengyuan Deng, Wenjie Zhao, Yan Chen, Qiaoniang Huang, Renliang Huang, Lifang Wen, Xiaoyang Yue, Frank Petersen, Xinhua Yu

**Affiliations:** ^1^Xiamen-Borstel Joint Laboratory of Autoimmunity, The Medical College of Xiamen University, Xiamen, China; ^2^Institute of Psychiatry and Neuroscience, Xinxiang Medical University, Xinxiang, China; ^3^Priority Area Asthma & Allergy, Research Center Borstel, Airway Research Center North (ARCN), Members of the German Center for Lung Research (DZL), Borstel, Germany

**Keywords:** primary Sjögren's syndrome, mouse model, presymptomatic feature, MHC II ectopic expression, TiterMax®, adjuvant

## Abstract

Ectopic expression of MHC II molecules on glandular cells is a feature of primary Sjögren's syndrome (pSS). However, the cause of this ectopic expression and its potential role in the pathogenesis of the disease remains elusive. Here, we report that ectopic expression of MHC II molecules on glandular cells represents an early presymptomatic event in a mouse model of pSS induced by immunization of Ro60_316-335 peptide emulsified in TiterMax® as an adjuvant. Ectopic expression of MHC II was induced by TiterMax® but not by complete freund's adjuvant (CFA). Furthermore, immunization with Ro60_316-335 peptide emulsified in TiterMax®, but not in CFA, induced a pSS-like disease in mice. Our results suggests that ectopic expression of MHC II molecules on glandular cells represents a presymptomatic feature of pSS and that such ectopic expression can be induced by exogenous factors. In addition, this study also provides a novel mechanism how adjuvants can amplify immune responses.

## Introduction

Primary Sjögren's syndrome (pSS) is a systemic autoimmune disorder mainly targeting salivary and lacrimal glands (1). Clinically, pSS is characterized by hypofunction of salivary and lacrimal cells leading to xerostomia (dry mouth) and xerophthalmia (dry eyes) (1). Histologically, pSS is featured by lymphocytic foci in the salivary and lacrimal glands (2) as well as ectopic expression of MHC II molecules on the glandular epithelial cells of those glands (3). In addition, autoantibodies including anti-SSA/Ro and anti-SSB/La autoantibodies, rheumatoid factor and anti-nuclear antibodies (ANA) are also hallmarks for pSS (4). Despite to the well-known clinical, histological, and immunological features of the symptomatic phase of pSS, little is known about the presymptomatic features of the disease. Currently, only autoantibodies such as anti-Ro60, anti-Ro52, and anti-La were described by Jonsson et al. in a large retrospective study (5) to be present in the presymptomatic phase of the disease.

Animal models are invaluable tools for the identification of relevant presymptomatic events in human autoimmune diseases (6, 7). With regard to pSS, presymptomatic events have been observed Non-obese diabetic (NOD) mice which develop a pSS-like disease spontaneously over time (8). Studies in this mouse strain revealed that the development the pSS-like disease consists of three sequential steps (9, 10). The first step is represented by intrinsic abnormalities in the exocrine glands, e.g., glandular cell apoptosis, followed by lymphocytes infiltration in the exocrine glands resulting in an impairment in secretion of saliva and tears. These observations in NOD mice suggest that intrinsic abnormalities in the exocrine glands and lymphocytic infiltration represent respective early and late presympomatic features in this mouse model.

Recently, we have established a novel mouse model for pSS by immunizing mice with human Ro60_316-335 peptide emulsified in TiterMax® as adjuvant (11). In this model, susceptible mice are characterized by generation of autoantibodies, lymphocytic infiltration and a decrease in tear secretion. By investigating gene expression profiles of lacrimal glands, we here aimed to identify the presymptomatic features of this novel mouse model of pSS.

## Methods and materials

### Mice and immunization

Female Balb/c mice were purchased from Shanghai SLAC Laboratory Animal Co., Ltd (Shanghai, China). All mice were housed under the specific pathogen free condition in the animal facility of Xiamen University. Immunization was performed using the protocol described previously (11). Briefly, female Balb/c mice were immunized with Ro60_316-335 peptide or PBS control emulsified in the TiterMax® (Alexis Biochemicals, Lorrach, Germany) or complete Freund's adjuvant (CFA). Protocols of all animal experiments were approved by the Institutional Animal Care and Use Committee of Xiamen University.

### Measurement of tears and saliva

Tears and saliva of mice were measured at week 0, 6, and 12 after immunization and further normalized to the bodyweight as described previously (11). Briefly, mice were starved for 16–18 h before the measurement, deeply anesthetized, and stimulated with pilocarpine hydrochloride (Sigma-Aldrich). Saliva was collected with a sponge immediately after the injection of pilocarpine for a time period of 20 min while tears were collected by using Phenol Red Thread (Jingming Ltd. Tianjin, China) at 10 min and 20 min time points after injection of pilocarpine. Both saliva and tear secretion volumes were normalized to individual mouse body weight.

### Detection of autoantibodies in sera

Anti-Ro60_316-335 autoantibodies in murine sera were detected by ELISA as described previously (11). In brief, SSA peptides were absorbed onto Costar EIA/RIA Plates (Corning Icorporated, corning, NY, USA), washed and blocked with 3% BSA in PBS supplemented with 0.05% Tween-20 (PBS-T), incubated with the respective mouse sera (1:200 dilution), and further washed with PBS-T. Bound antibodies were detected by using peroxidase conjugated goat anti-mouse IgG antibodies (Sigma, USA) and tetramethylbenzidine (Solarbio, Beijing, China) as substrate.

### Histopathological assessment

Twelve weeks after immunization, mice were sacrificed and tissues were collected for histopathological evaluation. Histology of salivary and lacrimal glands was evaluated after Haematoxylin and Eosin (H&E) staining of 5-μm-thick sections derived from paraffin embedded tissue. Cryosections from salivary and lacrimal glands were used for direct or indirect immunofluorescence and immunohistochemical staining. Direct immunofluorescence staining was performed for the detection of CD4^+^ T cell, CD8^+^ T cell, CD11c^+^ Dendritic cell and MHC II molecules by using Alexa-488 conjugated rat-anti-mouse CD4 (clone: RM4-5, Biolegend), Alexa-488 conjugated rat-anti-mouse CD8 (clone: 53-6.7, Biolegend), Alexa-488 conjugated Armenian Hamster-anti-Mouse CD11c antibody (clone: N418, Biolegend), and rat-anti-mouse I-A/I-E Antibody (clone: M5/114.15.2, Biolegend), respectively. Indirect Immunofluorescence staining was performed for the analysis of CD3^+^ T cell and CD19^+^ B cell by using rat-anti-mouse CD3 (clone: 17A2, Biolegend) and rat-anti-mouse CD19 (clone: 6D5, Biolegend), respectively, followed by using Alexa-488 conjugated goat-anti-rat IgG (clone: Poly4054, Biolegend). Immunohistochemistry was performed for the detection of MHC II molecules on lacrimal gland tissue cells by using rat-anti-mouse I-A/I-E antibody (clone: M5/114.15.2, Invitrogen) on cryosections.

### Gene expression profiling analysis

Total RNA was isolated from murine lacrimal glands at week 0, 2, and 6 after immunization using TRIzol Plus RNA Purification Kit (Invitrogen). The extracted total RNA from each sample was quantified by a NanoDrop ND-1000 spectrophotometer and RNA integrity was assessed by standard denaturing agarose gel electrophoresis. The double strand cDNA was synthesized by Invitrogen Superscript ds-cDNA synthesis kit (Invitrogen, USA) according to the manufacturer's protocol. cDNA was labeled with Cy3 using NimbleGen one-color DNA labeling kit (Roche NimbleGen Inc., USA). Labeled ds-cDNA samples were hybridized to NimbleGen Mouse 12x135K Gene Expression Microarray (Roche NimbleGen Inc., USA). Array scanning was done by the Axon GenePix 4000B microarray scanner (Molecular Devices Corporation, USA).

TIFF-files of scanned images were imported into NimbleScan software (version 2.5) for grid alignment and expression data analysis. Raw expression data were normalized through quantile normalization and the Robust Multichip Average (RMA) algorithm included in the NimbleScan software. Probe level (^*^_norm_RMA.pair) files and gene level (^*^_RMA.calls) files were generated after normalization. The gene level files were imported into Agilent GeneSpring GX software (version 11.5.1) for further analysis. Genes with values higher than or equal to lower cut-off: 100.0 in at least 3 out of 15 samples were chosen for further data analysis. Statistically significant differentially expressed genes (DEGs) were identified by Volcano Plot filtering (fold change ≥ 2.0, *p*-value ≤ 0.05). KEGG pathway analysis and Gene Ontology (GO) term enrichment analysis were applied to determine the roles of DEGs in various biological pathways and GO terms. To analyze the gene co-expression network, DEGs were submitted to the STRING database (version 10.5) for construction of a gene co-expression network. The constructed gene network was further edited by using the Cytoscape software platform (version: 3.6.1) (12).

### Real time quantitative PCR

Total RNA was isolated and processed by real time quantitative PCR (RT-qPCR) for gene expression analysis. PCR was performed by the use of an Applied Biosystems 7500 Real-Time PCR System (Applied Biosystems). The β*-actin (Actb)* gene was used as internal control. Data was normalized using the 2^−ΔΔ*Ct*^ method. A list of primers used for the RT-qPCR was presented as Supplementary Table 5.

### Statistical analysis

Except gene expression profiling data, all data were analyzed by using GraphPad Prism software (GraphPad Software Inc.). *P*-values below 0.05 were considered as statistically significant.

## Results

### Balb/c mice are susceptible to Ro60_316-335 peptide-induced pSS-like disease

**Figure 1:**
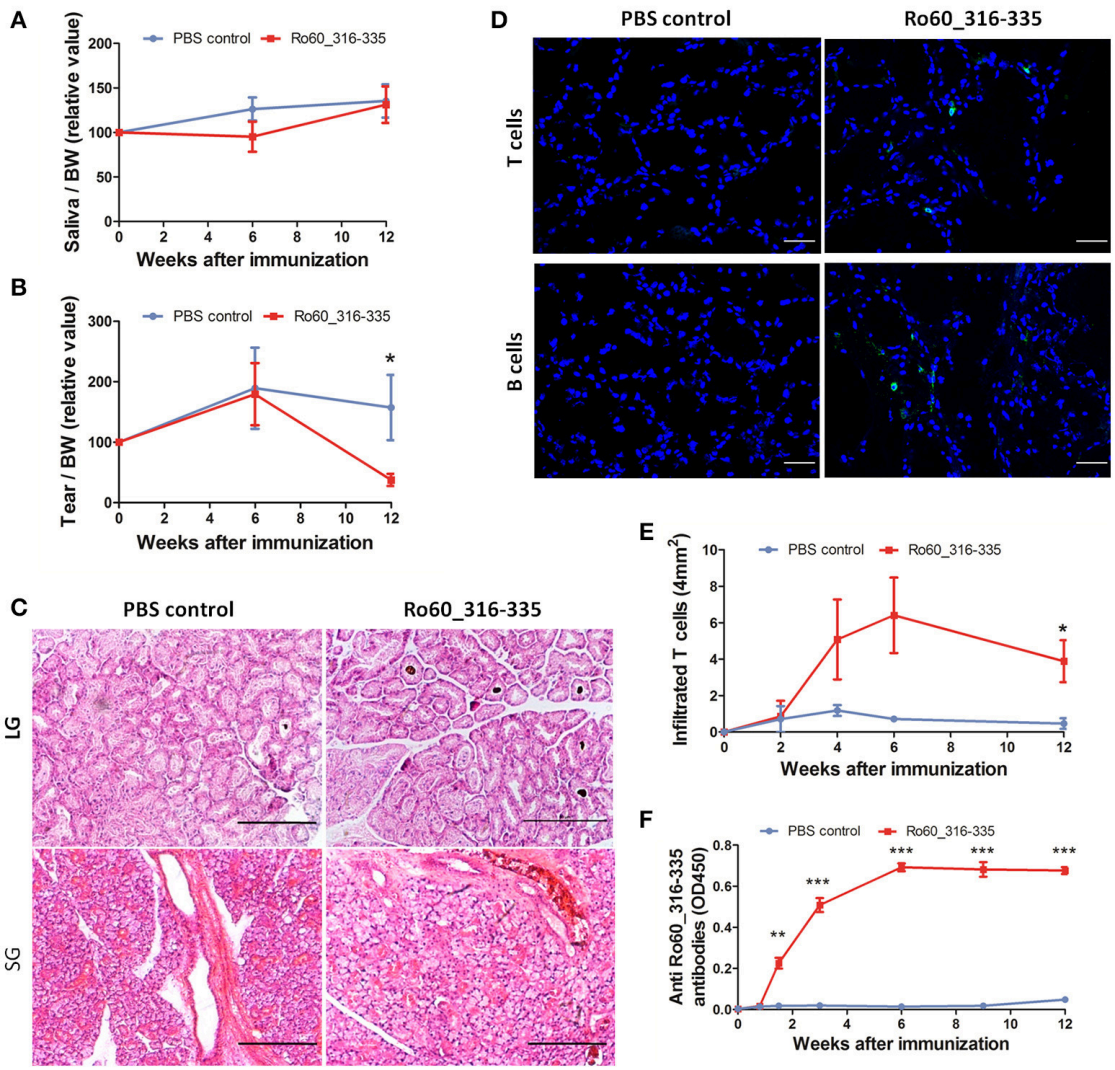
Balb/c mice are susceptible to Ro60_316-335-induced pSS-like disease. Balb/c mice were treated with Ro60_316-335 peptide either emulsified in TiterMax® or PBS. Secretion of saliva **(A)** and tears **(B)** was determined after pilocarpine stimulation. Values were normalized to the respective body weights and subsequently to the levels of secretion determined before immunization. Results of two experiments were pooled and data presented as Mean ± SEM, statistically significant differences between peptide-immunized mice (*n* = 11) and controls (*n* = 10) were calculated by using Student's *t*-test (*p < 0.05). **(C)** Representative micrographs after H&E staining of sections derived from paraffin embedded lacrimal glands (upper) and salivary glands (lower) of mice immunized with Ro60_316-335 peptide or PBS control. Bars, 100um. **(D)** Representative immunofluorescence micrographs CD3^+^ T cells (upper) and CD19^+^ B cells (lower) in lacrimal glands of Ro60_316-335- or PBS-treated mice. Bars, 50um. **(E)** Time-kinetics of CD3^+^ T cell infiltration in lacrimal glands of peptides immunized mice (*n* = 4) and controls (*n* = 4) at week 0, 2, 4, 6, and 12 after immunization. Data are presented as mean ± SEM, statistically significant differences between peptide- and PBS-treated mice were calculated by using Mann Whitney test (*p < 0.05). **(F)** Time-kinetics of anti-Ro60_316-335 autoantibodies production in the sera of Ro60_316-335 peptides immunized mice (*n* = 13) and controls (*n* = 11). Data presented as Mean ± SEM, statistically significant differences between peptide- and PBS- treated mice were calculated by using Mann Whitney test (**p < 0.01, ***p < 0.001).

Previously, we have demonstrated that development of Ro60_316-335 peptide-induced pSS-like disease in mice depends on the genetic background of the mice, where C3H/He mice were found to be susceptible but DBA/1 and C57BL/6 mice were shown to be resistant to the disease (11). Here, we investigated whether Balb/c mice are susceptible to experimental pSS. As shown in Figure 1A, secretion of tears in mice immunized with Ro60_316-335 peptide emulsified in TiterMax® was significantly reduced as compared to mice immunized with PBS control at week 12 after immunization, while no reduction in secretion of saliva was observed (Figure 1B). Although no lymphocytic focus was observed in the histology of either salivary or lacrimal glands (Figure 1C), immunofluorescence staining revealed the infiltration of both CD3^+^ T cells and CD19^+^ B cells in lacrimal glands of Ro60_316-335-immunized mice but not in the control mice (Figure 1D). CD3^+^ T cells, but not CD19^+^ B cells, were also observed in the salivary glands of Ro60_316-335-immunized mice (Supplementary Figure 1). Analysis of the subtypes of T cells revealed that the infiltrated T cells in lacrimal glands were composed of both CD4^+^ and CD8^+^ T cells (Supplementary Figure 2). Collectively, Balb/c mice immunized with Ro60_316-335 peptide showed lymphocytes infiltration into the exocrine glands and impairment in tear secretion, demonstrating that this strain is susceptible to experimental pSS. Notably, immunization with a different peptide derived from Ro60 (Ro60_480-494) also emulsified in TiterMax® failed to induce corresponding disease symptoms (Supplementary Figure 3), indicating the specificity of this model for Ro60_316-335.

We next analyzed kinetics of T cell infiltration and autoantibody production in Ro60_316-335 peptide-immunized mice. As shown in Figure 1E, infiltration of CD3^+^ T cells into the lacrimal gland was observed first at week 4 and reached a peak at week 6 after immunization. Moreover, anti-Ro60_316-335 peptide autoantibodies became detectable at day 10 and peaked at week 6 after immunization (Figure 1F). Kinetics of autoantibody production, lymphocytic infiltration, and impairment of tear secretion demonstrate that from week 0 to week 6 after the first immunization was the presymptomatic phase in Balb/c mice in this mouse model.

We then determined whether glandular cell apoptosis, a presymptomatic feature in NOD mouse model for pSS, exists also in the Ro60_316-335 peptide-induced model in Balb/c mice. As shown in Supplementary Figure 4, obvious glandular cell apoptosis was detected in lacrimal gland of neither Ro60_316-335 peptide-immunized mice nor control mice, suggesting that apoptosis is not a feature of this model.

### Gene expression profiling of lacrimal glands from the presymptomatic phase of the disease

Although our previous study has shown that C3H/He mice are also susceptible to the Ro60_316-335 peptide induced pSS-like disease (11), the time frame of the presymptomatic phase in Balb/c mice is better defined than that in C3H/He mice. Therefore we investigated the presymptomatic features in the lacrimal glands in the Balb/c strain. We determined gene expression profiling of the lacrimal glands in the presymptomatic phase, including the status prior to immunization (week 0), at week 2 and 6 weeks after immunization with Ro60_316-335 peptide emulsified in TiterMax®. Comparison were performed for peptide-immunized mice vs. untreated mice (week 0), peptide-immunized mice vs. PBS/ TiterMax®-treated control mice, and PBS/ TiterMax®-treated control mice vs. untreated mice. As shown in Supplementary Figures 5, 6; Supplementary Tables 1, 2, each comparison resulted in hundreds of upregulated or downregulated genes. To further characterize those differentially expressed genes, we performed gene ontology (GO) enrichment analysis (Supplementary Table 3), pathway enrichment analysis (Supplementary Table 4), and gene network analysis.

**Figure 2:**
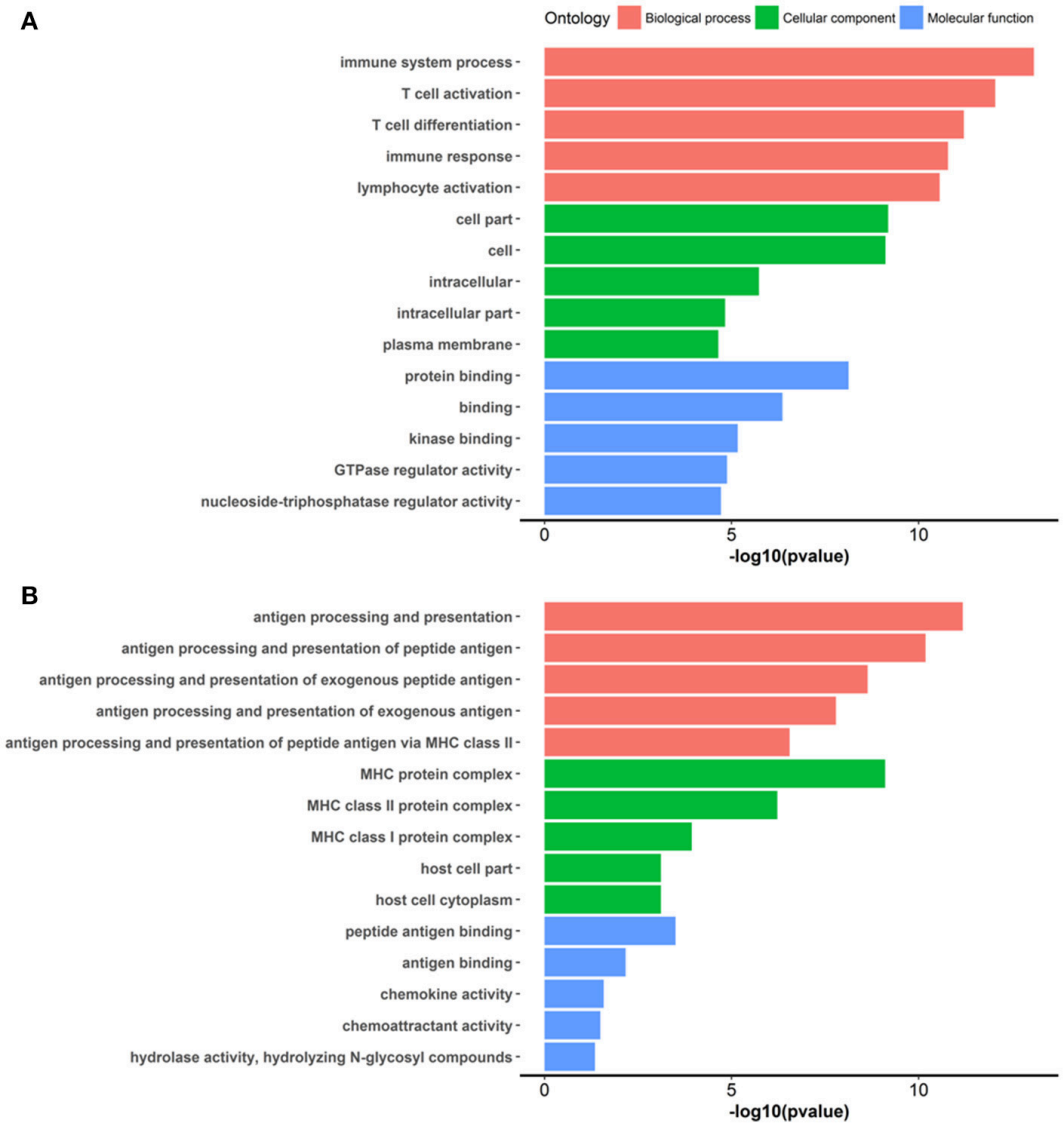
Gene ontology (GO) enrichment of differentially expressed genes (DEGs) in the lacrimal glands of mice. **(A)** GO terms enriched in genes up-regulated in lacrimal glands of mice at week 6 after immunization with Ro60_316-335 peptide compared to untreated mice (week 0). **(B)** GO terms enriched in genes up-regulated in lacrimal glands of mice at week 2 after immunization with Ro60_316-335 peptide compared to untreated mice. The top 5 significantly enriched GO terms in “biological process,” “cellular component,” and “molecular function” are presented. Names of the GO terms are indicated in Y axis, while the X axis indicates *p*-values.

**Figure 3:**
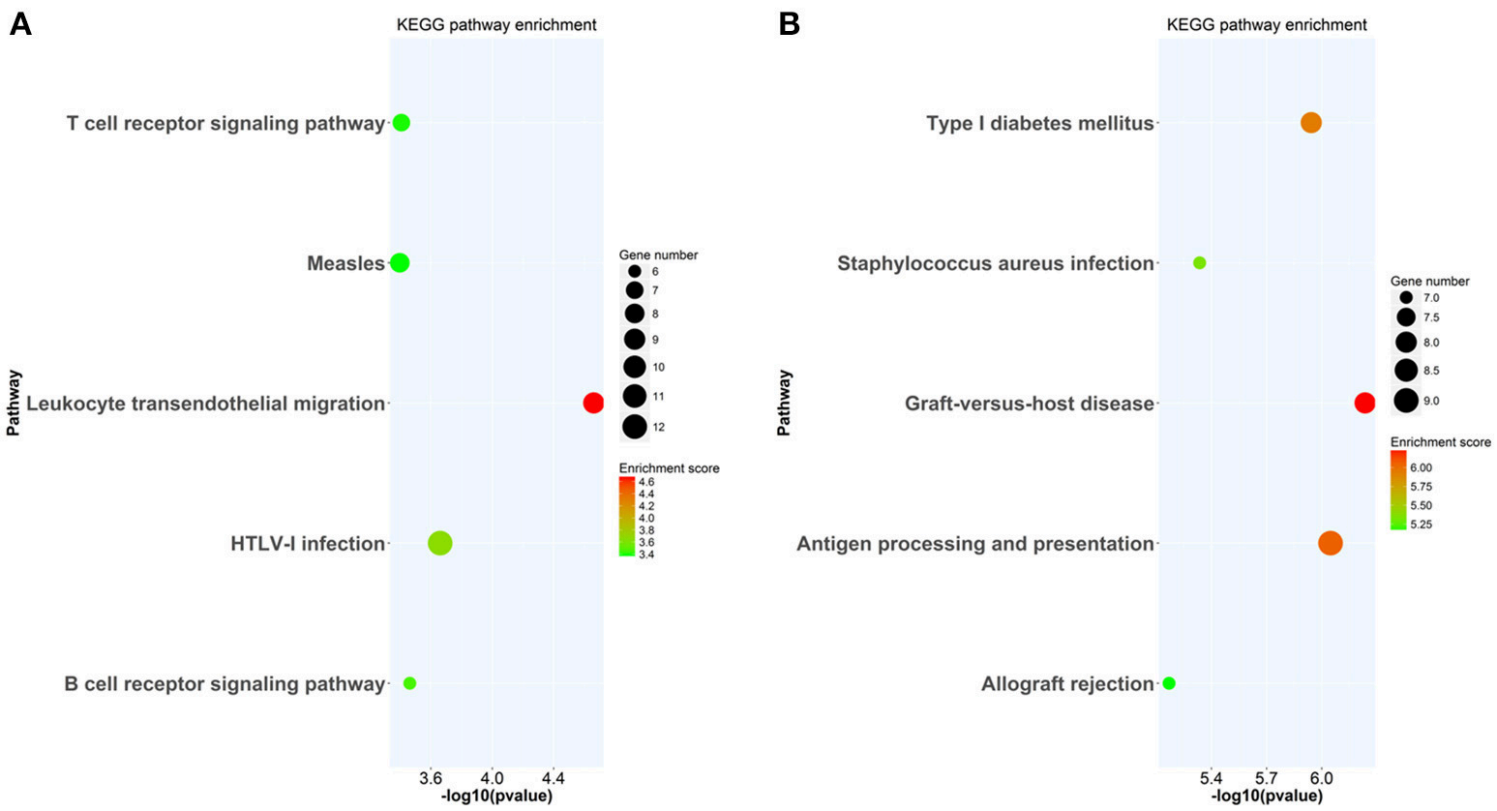
KEGG pathway enrichment analysis of differentially expressed genes (DEGs) in lacrimal glands of mice. **(A)** KEGG pathways enriched in genes up-regulated in lacrimal glands of mice at week 6 after immunization with Ro60_316-335 peptide compared to untreated mice (week 0). **(B)** KEGG pathways enriched in genes up-regulated in lacrimal glands of mice at week 2 after immunization with Ro60_316-335 peptide compared to untreated mice. The top 5 significantly enriched KEGG pathways are shown. HTLV-1, Human T-cell lymphotropic virus type 1.

**Figure 4:**
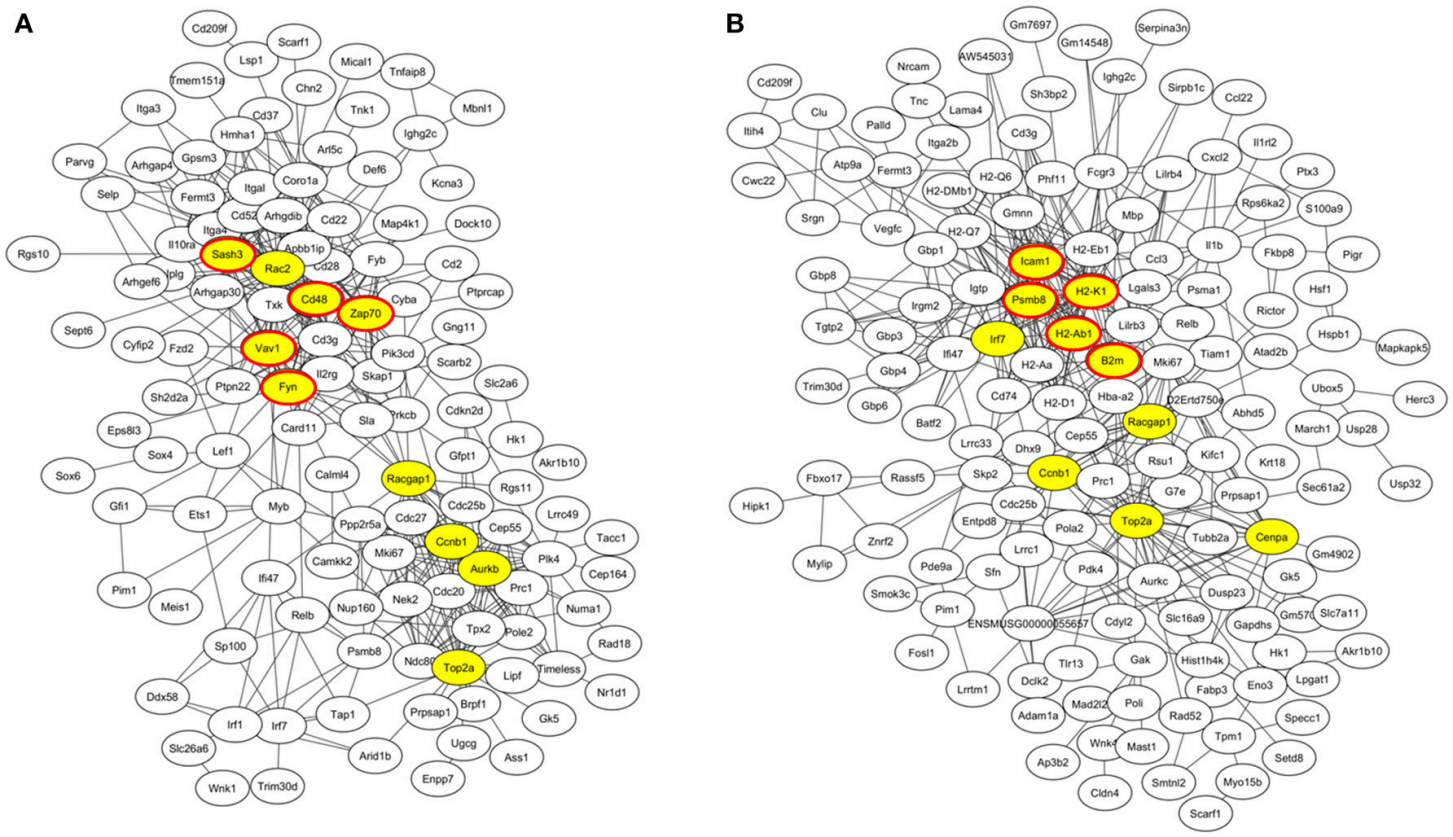
Gene co-expression network of differentially expressed genes (DEGs) in lacrimal glands of mice. **(A)** Gene network of genes up-regulated in lacrimal glands of mice at week 6 after immunization with Ro60_316-335 peptide compared to untreated mice (week 0). The top 10 connected genes are labeled in yellow. Among the top ten connected genes, genes involved in immune responses are highlighted with red cycles. **(B)** Gene network of genes up-regulated in lacrimal glands of mice at week 2 after immunization with Ro60_316-335 peptide compared to untreated mice. The top 10 connected genes are labeled in yellow. Among the top ten connected genes, genes involved in antigen presentation are highlighted with red cycles.

At week 6 after immunization, top enriched GO terms in upregulated genes in the lacrimal glands of Ro60_316-335 peptide-immunized mice as compared to untreated mice (week 0) belong to the gene groups of “immune system process,” “T cell activation,” “immune response,” and “lymphocyte activation” (Figure 2A). These results were in line with findings of the pathway enrichment analysis, where those upregulated genes were allocated in the pathways “leukocyte transendothelial migration,” “B cell receptor signaling,” and “T cell receptor signaling” (Figure 3A). Interestingly, two infection related pathways, measles, and human T-cell lymphotropic virus type 1 (HTLV-1) infection, also appeared in the pathway enrichment analysis, suggesting that the immunization mediated immune responses within lacrimal glands was similar to those against infections. Furthermore, a gene network constructed on the basis of the identified upregulated gene showed that 7 out of top 10 central nodes belong to genes involved in “immune responses” (Figure 4A). Therefore, all three bioinformatic analysis strategies demonstrate that immune response associated genes were upregulated in lacrimal glands of Ro60_316-335 peptide-immunized mice compared to untreated mice. These findings are supported by our observation that infiltration of lymphocytes in the lacrimal glands reached the peak at 6 weeks after immunization. Notably, those immune response associated genes upregulated in lacrimal glands of peptide-immunized mice at week 6 after immunization were not seen in PBS/ TiterMax®-treated control mice (Supplementary Tables 3, 4), suggesting that their upregulation was induced specifically by the peptide immunization.

**Figure 5:**
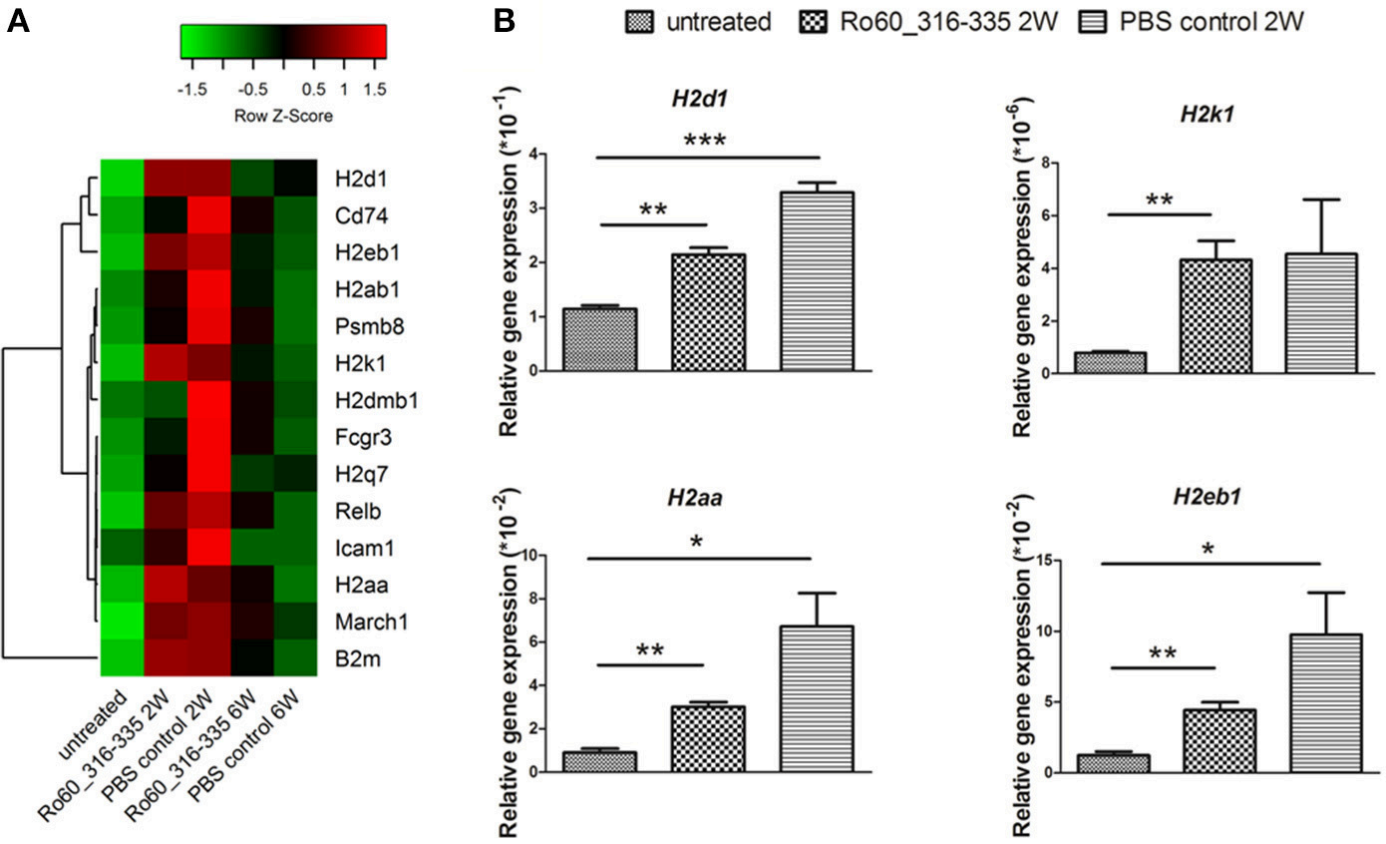
Expression of genes involved in antigen processing and presentation in lacrimal glands of mice. **(A)** Heatmap of differentially expressed genes involved in antigen processing and presentation, with green and red color representing low and high gene expression, respectively. Signal intensity of gene expression was normalized by using the z-score and indicated in color from green (low) to red (high). Calculation and plotting were performed by using package *heatmap.2* in RStudio. **(B)** Validation of gene expression of *H2d1, H2k1, H2aa*, and *H2eb1* in lacrimal glands of mice at week 2 after immunization with Ro60_316-335 peptide, PBS/Titermax® control, and in untreated mice (week 0). The quantification of gene expression was determined by real time quantitative PCR with β*-actin* gene expression as internal control for normalization. Data are presented as mean ± SEM, statistical differences between groups were determined by using Student's *t*-test (*p < 0.05, **p < 0.01 and ***p < 0.001).

Since gene expression data suggest that there were immune responses in the lacrimal glands of mice immunized with Ro60_316-335 peptide at 6 weeks after immunization, we next evaluated the gene expression profiling at 2 weeks after immunization in the early presymptomatic phase. As compared to week 0, top enriched GO terms of upregulated genes in lacrimal glands at week 2 after immunization with the Ro60_316-335 peptide were “antigen processing and presentation,” “MHC protein complex,” and “peptide antigen binding” (Figure 2B), suggesting that genes involved in antigen presentation and processing were activated. This notion was confirmed by pathway enrichment analysis and gene network analysis, which showed that “antigen processing and presentation” was one of the top enriched pathways (Figure 3B) and that 5 out of top 10 central nodes consist of genes involved in antigen presentation and processing (Figure 4B), respectively. Therefore, our bioinformatic analysis demonstrate that in the lacrimal glands of mice at week 2 after immunization genes related to antigen presentation and processing were upregulated. Surprisingly, these genes were also found to be upregulated in mice treated PBS/TiterMax® as a control (Supplementary Tables 3, 4), indicating that regulation to these genes was not driven by the antigen but by the adjuvant. Upregulated genes encompass several MHC I and MHC II genes, including *H2d1, H2eb1, H2ea1, H2k1, H2dmb1, H2q7, H2aa*, and *B2m* (Figure 5A). To confirm the upregulation of MHC genes, we performed real time quantitative PCR to determine the expression of *H2d1, H2k1, H2eb1*, and *H2aa*. As predicted, we found, all four MHC genes to be upregulated in the TiterMax®-treated groups in comparison to the respective untreated control group (Figure 5B).

### Ectopic expression of MHC II on murine lacrimal glands

**Figure 6:**
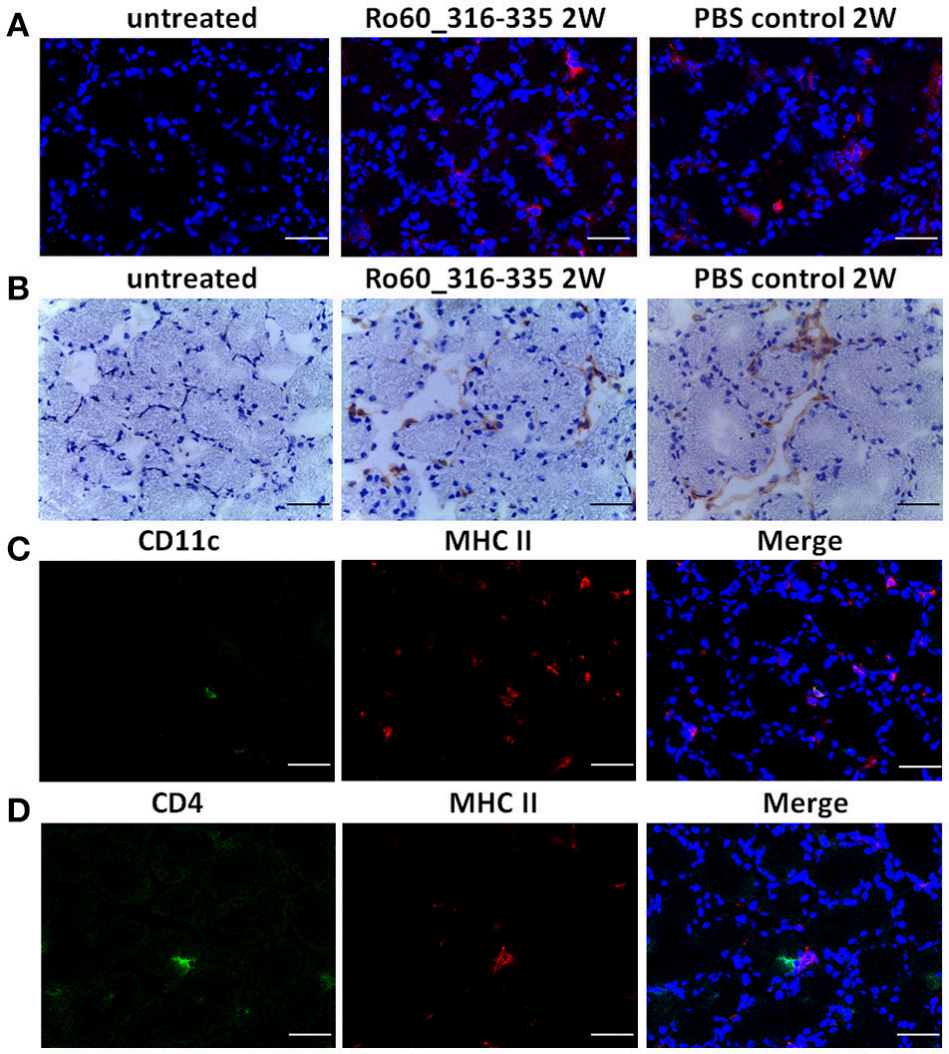
Ectopic expression of MHC II molecules on glandular cells in lacrimal glands of TiterMax®-treated mice. **(A)** Representative micrographs of MHC II molecules on lacrimal glands from mice immunized with Ro60_316-335 peptides or PBS emulsified in TiterMax® at the week 2 after immunization and on untreated mice (week 0). MHC II molecules were detected on cryosections of the lacrimal glands by direct immunofluorescence staining using Alexa-647 conjugated rat-anti-mouse I-A/I-E Antibody. Bars, 50um. **(B)** Representative micrographs of MHC II molecules on lacrimal glands of mice. Expression of MHC II molecules was determined by immunohistochemistry on cryosections. Bars, 50um. **(C)** Co-staining for CD11c and MHC II molecules on cryosections of lacrimal glands from mice immunized with Ro60_316-335 peptides. CD11c and MHC II molecules on lacrimal gland tissue cells were detected by using Alexa-488 conjugated Armenian Hamster-anti-Mouse CD11c antibody and Alexa-647 conjugated rat-anti-mouse I-A/I-E Antibody, respectively. Bars, 50um. **(D)** Co-staining for CD4 and MHC II molecules on cryosections of lacrimal glands from mice immunized with Ro60_316-335 peptides. CD4 and MHC II molecules on lacrimal gland tissue cells were detected by Alexa-488 conjugated rat-anti-Mouse CD4 antibody and Alexa-647 conjugated rat-anti-mouse I-A/I-E Antibody, respectively. Bars, 50um.

Under physiological condition, glandular cells express MHC I but not MHC II molecules. Thus, the upregulation of MHC II molecules observed here represent most likely an ectopic expression. Since such an ectopic expression of HLA II on epithelial cells of salivary glands is a feature of pSS patients (3, 13), we further characterized the expression of MHC II in lacrimal glands of the immunized mice. As expected, MHC II molecules were not expressed on glandular cells prior to immunization. However, ectopic expression of MHC II molecules was observed at week 2, 6 and 12 after treatment with TiterMax®, irrespective whether a further antigen was co-applied or not (Figure 6A and Supplementary Figure 7). Immunohistochemical staining revealed further that MHC II molecules were ectopically expressed on both lacrimal acinar and ductal cells in the lacrimal glands of TiterMax®-treated mice (Figure 6B). To confirm that elevated expression of MHC II molecules was associated with their upregulation on glandular cells but not by infiltration of professional APCs, we performed co-staining of tissue against MHC II in combination with CD11c. Although we could identify a small number of CD11c^+^MHCII^+^ APCs in the glandular tissue, the majority of MHC II^+^ cells in the lacrimal glands of TiterMax®-treated mice scored negative for CD11c (Figure 6C).

To investigate whether the ectopic expression of MHC II molecules is limited to tissues of lacrimal glands, we determine the expression of MHC II in other organs including salivary glands, lung, kidney and heart. As shown in Supplementary Figure 8, ectopic expression of MHC II molecules could be detected in all analyzed organs in mice treated with TiterMax® but not in untreated controls.

To further examine whether MHC II expressing glandular cells interact with infiltrated CD4^+^ T cells, we performed co-staining of MHC II molecules with CD4. As shown in Figure 6D, co-localization of CD4^+^ T cells and MHC II expressing cells was observed in the lacrimal glands of mice immunized with Ro60_316-335 peptide, indicating that glandular cells may have the ability to present antigen to corresponding T cells. Noteworthy, no such interaction was observed in mice which received TiterMax® in the absence of the peptide.

### CFA does not induce ectopic expression of MHC II molecules

**Figure 7:**
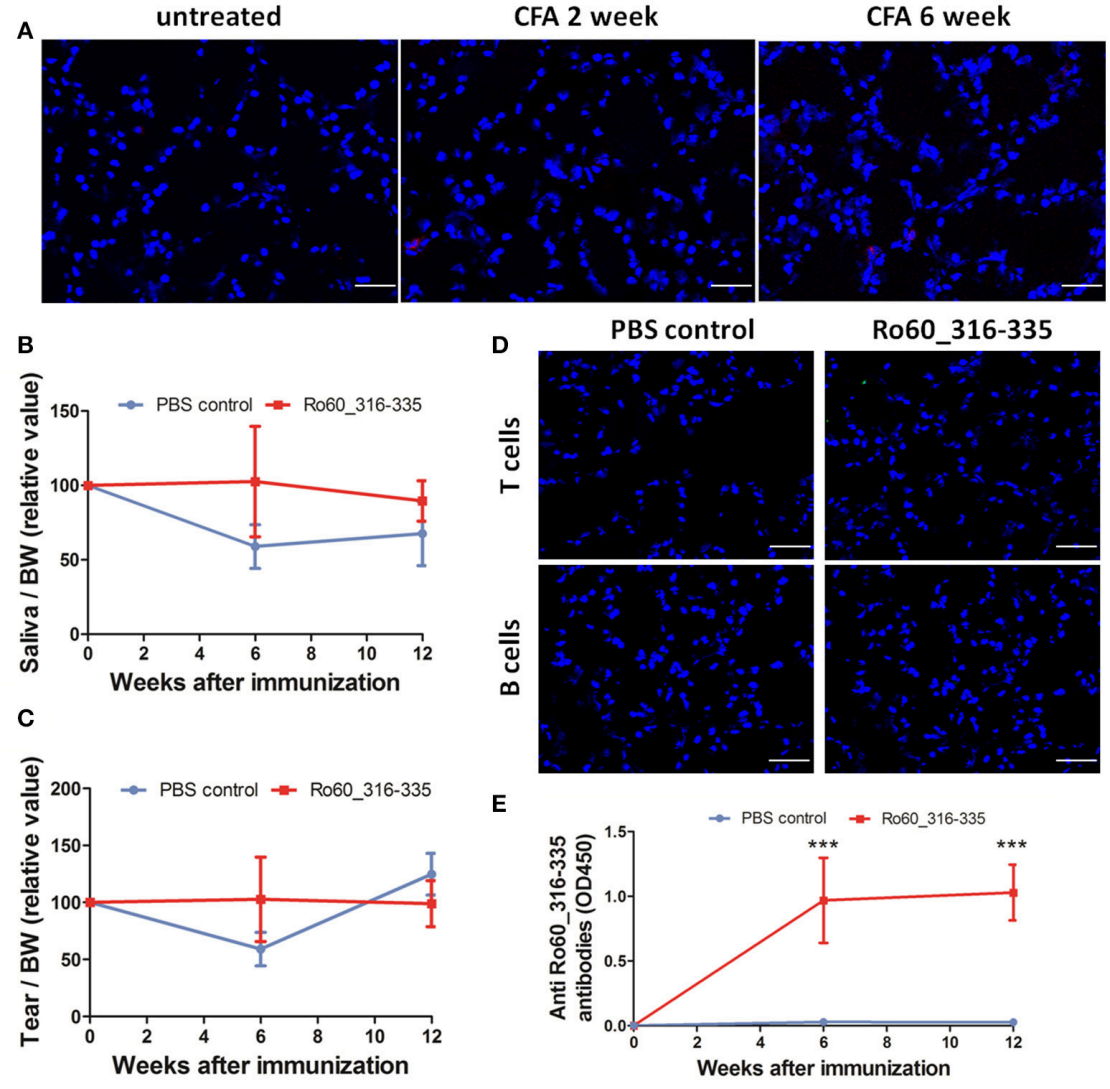
Immunization with Ro60_316-335 peptide emulsified in complete freund's adjuvant (CFA) failed to induce pSS-like disease in Balb/c mice. **(A)** Representative immunofluorescence micrographs of MHC II molecules on cryosections of lacrimal glands from mice treated with PBS emulsified in CFA at week 0, 2, and 6 after immunization. Bars, 50 μm. Secretion of saliva **(B)** and tears **(C)** was determined after pilocarpine stimulation. Values were normalized to the respective body weights and subsequently to the levels of secretion determined before immunization. Data presented as mean± SEM, statistically significant differences between peptide-immunized mice (*n* = 13) and controls (*n* = 8) were determined using Student' *t*-test. **(D)** Representative immunofluorescence micrographs of CD3^+^ T cells (upper) and CD19^+^ B cells (lower) in lacrimal glands of Ro60_316-335-immunized mice or PBS-treated control mice. Bars, 50 μm. **(E)** Production of anti-Ro60_316-335 antibodies in the sera of mice immunized with Ro60_316-335 or PBS emulsified in CFA. Data are presented as mean ± SEM, statistically significant differences between peptide- and PBS- immunized mice were calculated by using Mann Whitney test (***p < 0.001).

Finally, we then investigated whether the adjuvant induced ectopic expression of MHC II molecules is specific for TiterMax® or a more general effect which can be mediated by other adjuvants too. However, treatment with CFA did not induce the ectopic expression of MHC II molecules in the lacrimal glands (Figure 7A) in Balb/c mice. Furthermore, immunization with Ro60_316-335 peptide emulsified in CFA failed to induce a pSS-like disease. Although these mice did produce autoantibodies against Ro60_316-335 peptide (Figure 7E), neither impairment in tears secretion (Figures 7B,C) nor lymphocytic infiltration (Figure 7D) was observed.

## Discussion

In this study, we could demonstrate that ectopic expression of MHC II molecules in lacrimal glands represents an early presymptomatic event in the Ro_316-335 peptide-induced model for pSS. Given that adjuvants are able to enhance maturation of dendritic cells and to increase antigen presentation, it is not surprising that application of an adjuvant mediates the upregulation of MHC molecules on professional APCs (14). However, the adjuvant-induced ectopic expression of MHC II molecules on glandular epithelial cells has not been observed so far. To our knowledge, this study reports for the first time on the ectopic expression of MHC II molecules as a presymptomatic feature of animal models of pSS.

In 1985, Lindahl et al. reported that HLA_DR molecules are ectopically expressed on the minor salivary gland epithelial cells around the dense lymphocytic infiltrates in pSS patients (13). Subsequent *in-situ* histological studies demonstrated that both salivary acinar and ductal cells ectopically express MHC II molecules (3, 15). However, it is not clear whether this ectopic expression is a presymptomatic feature or already a consequence of the disease manifestation. So far, the only reported evidence for an ectopic expression of MHC II in an animal models for pSS derives from observations in *RbAp48* transgenic mice. In 2008, Ishimaru et al. reported that these mice develop a pSS-like disease spontaneously which is associated with an ectopic expression of MHC II molecules on glandular epithelial cells (16). Since in this study only the histology of exocrine gland of diseased mice was evaluated, it is not clear whether this ectopic expression of MHC II molecules is a presymptomatic feature of this model. By contrast, we here demonstrate that ectopic expression of MHC II molecules on both acinar and ductal cells is a true presymptomatic feature of Ro60_316-335 peptide-induced mouse model for pSS.

Although ectopic expression of MHC II molecules on glandular epithelial cell is a well-defined feature of pSS (13), it is not clear whether this abnormality is caused by a genetic dysregulation or by exogenous factors. In the *RbAp48* transgenic mouse model, ectopic expression of MHC II molecules is a clear consequence of a genetic modification and is mediated by the overepression of the *RbAp48* gene (16). In our study, ectopic expression of MHC II molecules is induced by application of TiterMax®, suggesting that the ectopic expression can be caused by an exogenous factor. Taken together, evidence from animal studies suggests that both genetic and environmental factors can mediate the ectopic expression of MHC II molecules on glandular epithelial cells in pSS.

Expression of MHC II genes are controlled by interferon regulatory factor 1 (IRF-1) and Class II Major histocompatibility complex transactivator (CIITA) as primary regulators of MHC II expression (17, 18). Moreover, it has been reported that IFN-γ regulate the expression of MHC II by acting on IRF-1 and CIITA (19). Consistently, IRF-1 has been reported to be highly expressed in the salivary gland from pSS patients (20), supporting the hypothesis that the ectopic expression of MHC II is regulated by IRF-1. With regard to the ectopic expression of MHC II on glandular epithelial cells in mice, Ishimaru et al. could demonstrate the production of IFN-γ by salivary gland epithelial cells which induces the upregulation of IRF-1 and CIITA and further leads to the ectopic expression of MHC II molecules in exocrine glands of the *RbAp48* transgenic mice (16). In line with these findings, we observed a significant upregulation of *IRF-1* and *CIITA* gene expression in the lacrimal gland of TiterMax® treated mice as compared to untreated mice (Supplementary Figure 9), providing indirect evidence that ectopic expression of MHC II molecules is likewise mediated by upregulation of these two molecules.

Since the MHC II molecules play a pivotal role in antigen presentation and subsequent CD4^+^ T cell activation (21), it is conceivable that glandular epithelial cells expressing MHC II molecules might act as APCs and thus are involved in the initiation of the disease manifestation. This notion is supported by our observation that infiltrated CD4^+^ T cells co-localized with glandular cells ectopically expressing MHC II molecules. Previously, Ishimaru et al. showed that salivary gland epithelial cells (SGEC) obtained from pSS patients express both MHC II molecules and co-stimulatory cytokines and are able to mediate the initiation, development and maintenance of inflammatory response as non-professional APCs *in vitro* (22), supporting a role of MHC II-expressing glandular epithelial cells as APCs.

Interestingly, TiterMax®, but not CFA, can induce ectopic expression of MHC II molecules. Furthermore, only TiterMax® but not CFA can be used as the effective adjuvant for Ro60_316-335 peptide-induced mouse model of pSS. This observation suggests that these two adjuvants are different in their actions in triggering immune responses, especially in triggering innate immune responses. Studies in which both adjuvants were compared have demonstrated that CFA is able to mediate stronger immune response than TiterMax® (23–26), in terms of antibody production and chronicity of inflammation. However, the difference between CFA and TiterMax® in triggering innate immune responses was not clear so far.

Adjuvants are effective means to potentiate cellular and humoral immune responses in modeling human immune related diseases. The mode of action of adjuvants includes prolongation of the lifetime of (auto-)antigens, enhancement of antigen delivery to APC, and stimulation of the innate immune system (14, 27). The current study demonstrates that TiterMax® is able to induce ectopic expression of MHC II molecules on epithelial cells. Previously, Ishimaru and colleagues have shown that SGEC of pSS patient express beside MHC II molecules also antigen presenting-associated molecules such as CD86, CD80, and ICAM-1 (16). Moreover, SGEC are able to activate CD4^+^ T cells, suggesting that they can function as antigen presenting cells (16). Therefore, the findings of the current study suggests a novel mechanism of adjuvant-mediated activation of the adaptive immune response by converting epithelial cells into APCs.

Although our results provide an idea on presymptomatic processes in experimental pSS, some limitations in this study have to be discussed. First, the Ro60_316-335 peptide-induced disease phenotypes in Balb/c mice are rather mild, which makes this model not ideal for all studies on this disease. Second, lymphocytic foci in the exocrine glands, a feature of pSS patients, are not present in Balb/c mice and only a rather mild infiltration of lymphocytes could be detected in lacrimal and salivary glands. It is not clear whether this limited number of infiltrated lymphocyte and their interaction with glandular cells are relevant to the development of disease. Third, given that both lacrimal and salivary glands are characterized by mild lymphocytic infiltration and MHC II ectopic expression, it is unclear why only the secretion of tears is impaired. One possible explanation for this could be discrepant susceptibilities of both glands to autoimmune-mediated impairment. This idea is supported by observations in two other murine models of pSS. In thrombospondin-1-deficient mice impairment in tear secretion occurs without effect on the production of saliva (28) and in transgenic mice overexpressing retinoblastoma-associated protein 48 a more severe impairment of lacrimal gland function was seen than in the function of salivary glands (16). Finally, since TiterMax® represents a multi-component reagent, the identity of the defined substance relevant for the ectopic expression of the MHC II molecules has to be clarified.

In conclusion, the current study shows that TiterMax®-induced ectopic expression of MCH II molecules on exocrine glands is an important feature in the early presymptomatic phase of the Ro60 peptide-induced mouse model of pSS. These findings suggest that that ectopic expression of MHC II molecules in the exocrine glands might be a presymptomatic feature of pSS and such ectopic expression can be induced by exogenous factors. Furthermore, this study also suggests a novel mechanism of adjuvant action in potentiating immune responses.

## Author contributions

JY, JZ, FD, WZ, YC, QH, RH, LW, XYue performed experiments, XYu conceived and supervised the project, JY, XYu, and FP wrote the manuscript.

### Conflict of interest statement

The authors declare that the research was conducted in the absence of any commercial or financial relationships that could be construed as a potential conflict of interest.
