# Supplementary material for: Gene Expression Profiling of Lacrimal Glands Identifies the Ectopic Expression of MHC II on Glandular Cells as a Presymptomatic Feature in a Mouse Model of Primary Sjögren's Syndrome
Source: Front Immunol. 2018 Oct 31;9:2362. doi: 10.3389/fimmu.2018.02362 (PMC6220427; doi:10.3389/fimmu.2018.02362)
Supplement: Supplementary file 5 [file Data_Sheet_1.PDF]

Supplementary information

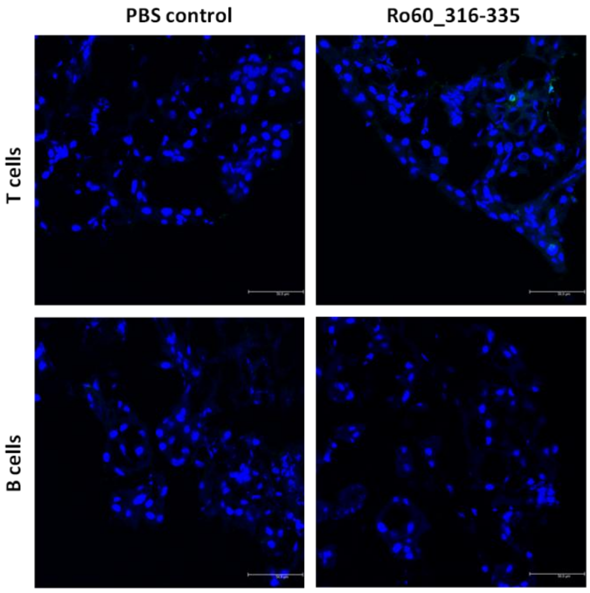

Supplementary figure 1. Infiltration of lymphocytes in the salivary glands of mice. Representative immunofluorescence micrographs of $CD3^+$ T cells (upper panel) and $CD19^+$ B cells (lower panel) in salivary glands of mice immunized with Ro60_316-335 peptide or PBS control. T cells and B cells were determined on cryosections by using rat-anti-mouse CD3 and rat-anti-mouse CD19 antibodies, respectively. Bars, 50 µm.

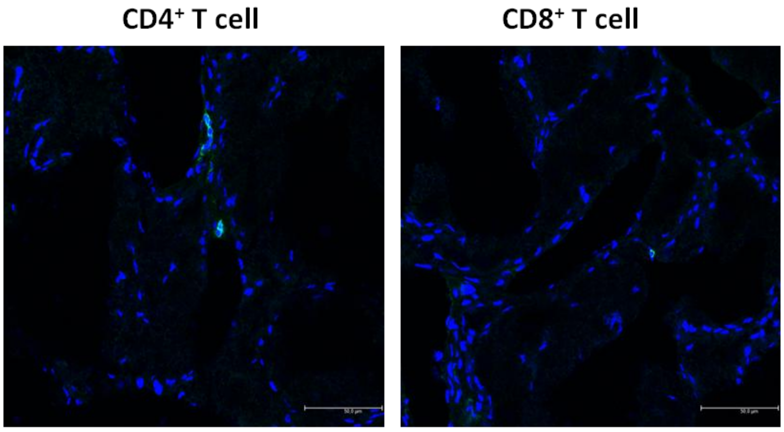

Supplementary figure 2. Subtypes of infiltrated T cells in lacrimal glands of mice. Representative immunofluorescence micrographs of $CD4^+$ T cells (left) and $CD8^+$ T cells (right) in lacrimal glands of Ro60_316-335-immunized mice. $CD4^+$ T cells and $CD8^+$ T cells were determined on cryosections by using Alexa488 conjugated rat-anti-mouse CD4 and Alexa488 conjugated rat-anti-mouse CD8 antibodies, respectively. Bars, 50 µm.

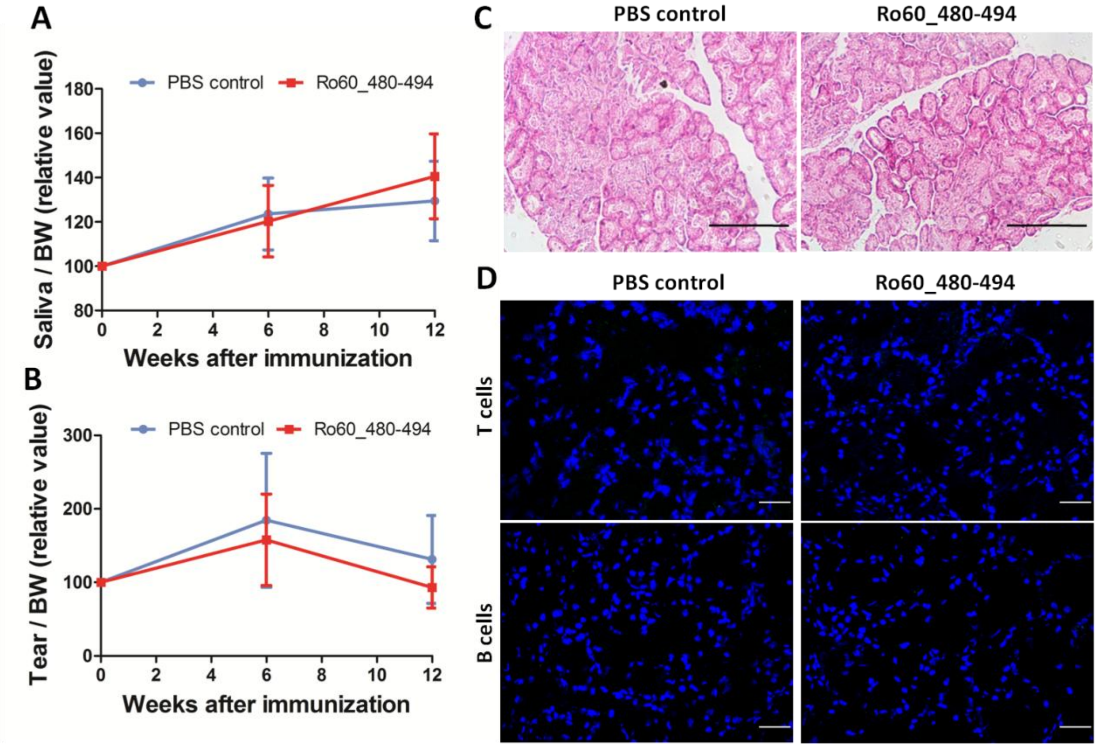

Supplementary figure 3. Immunization with Ro60_480-494 peptide failed to induce a pSS-like disease. Balb/c mice were treated with Ro60_480-494 or PBS (control) emulsified in Titermax. Secretion of saliva (A) and tears (B) was determined after pilocarpine stimulation. Values were normalized to the respective body weights and subsequently to the levels of secretion determined before immunization. Data are presented as mean ± SEM, statistically significant differences between peptide-immunized mice (n=9) and controls (n=7) were calculated by using Mann Whitney test. C: Representative micrographs of paraffin sections of lacrimal glands after H&E staining derived from the mice immunized with Ro60_480-494 peptide or PBS (control). Bars, 100µm. D: Representative immunofluorescence micrographs of $CD3^{+}$ T cells (upper panel) and $CD19^{+}$ B cells (lower panel) in lacrimal glands of Ro60_480-494- or PBS-(control) treated mice. Bars, 50 µm.

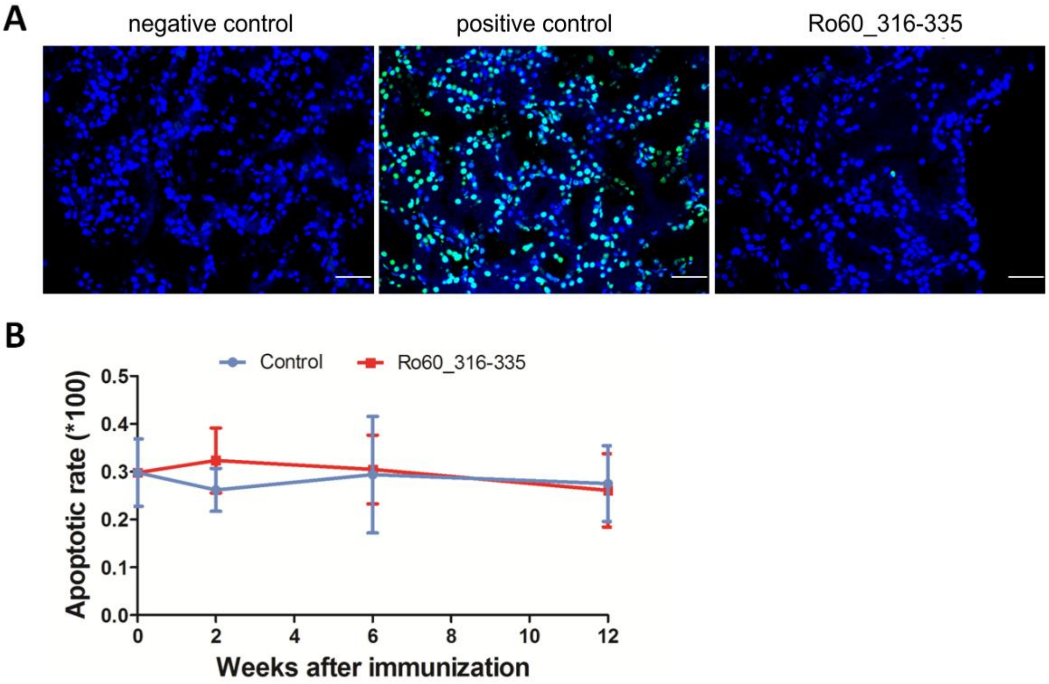

Supplementary figure 4: Apoptosis in the lacrimal glands of mice. Apoptotic cells were detected on paraffin sections of lacrimal glands by using DeadEnd™ Fluorometric TUNEL System. A: Representative micrographs of apoptotic cells. In negative controls samples were incubated without rTdT (Recombinant Terminal Deoxynucleotidyl Transferase), positive controls were generated by digestion of samples with DNase I. Bars, 50µm. B: Time-kinetics of apoptosis in lacrimal glands of mice immunized with Ro60_315-336 peptide (n=3) or treated with PBS (control) (n=3) emulsified in Titermax. Apoptotic rates were calculated as ratio between the number of apoptotic cells and the total number of cells. Cells were counted by using ImageJ software. Data was presented as mean ± SEM. Statistic differences between peptide-immunized mice and control samples were analyzed by Student's t test.

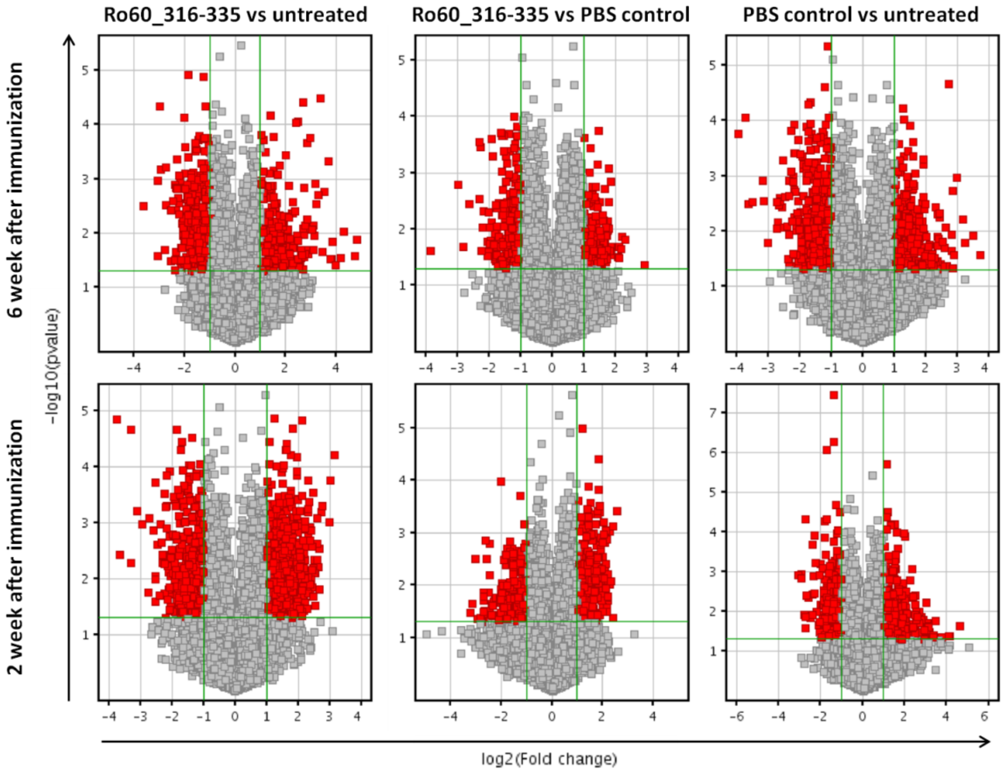

Supplementary figure 5: Comparison of differentially expressed genes (DEG) in murine lacrimal glands by volcano plot representation. DEGs identified in lacrimal glands of mice at week 6 (upper panel) or week 2 (lower panel) after immunization. DEGs were identified by comparing gene expression in mice immunized with Ro60_316-335 peptide with non-immunized mice (left), mice immunized with Ro60_316-335 peptide with mice treated with PBS (control) (middle), and mice treated with PBS (control) with non-immunized mice (right). The y-axis corresponds to the significance level of $\log_{10}$ (pvalue), and the x-axis displays the log2 fold change value. Red dots represent significantly differential expressed genes ($p < 0.05$, fold change > 2).

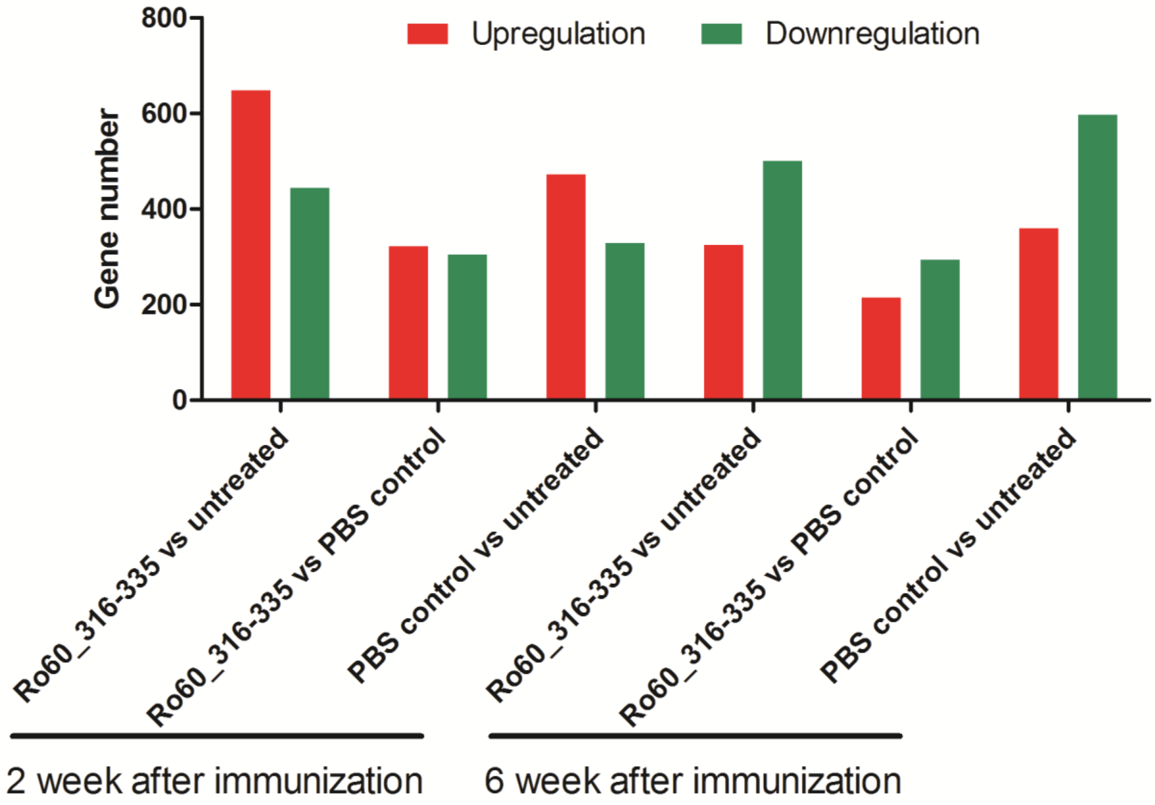

Supplementary figure 6: Comparison of the numbers of differentially expressed genes (DEG) in murine lacrimal. Data derived from the experiment described in the legend to Supplementary figure 5 were further analyzed according to the numbers of DEG. The y-axis displays the number of up-regulated or down-regulated genes, and the x-axis indicates the compared groups.

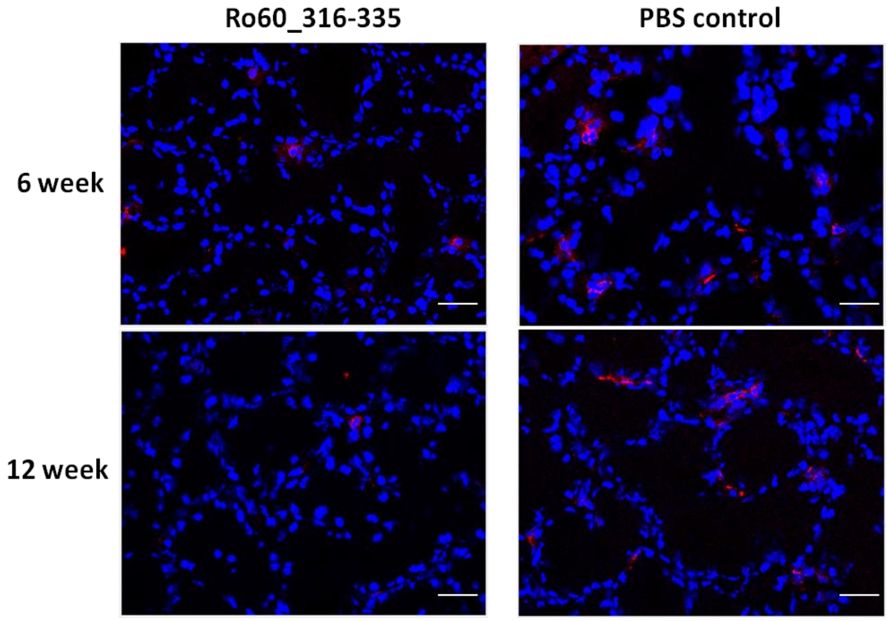

Supplementary Figure 7. Ectopic expression of MHC II molecules in lacrimal glands at week 6 and 12 after immunization. Mice treated with Ro60_316-335 peptide or PBS (control) emulsified in Titermax were sacrificed at week 6 or 12 after immunization. Ectopic expression of MHC II molecules on lacrimal glands were detected by immunofluorescence staining and the representative micrographs are shown. Bars, 50 µm.

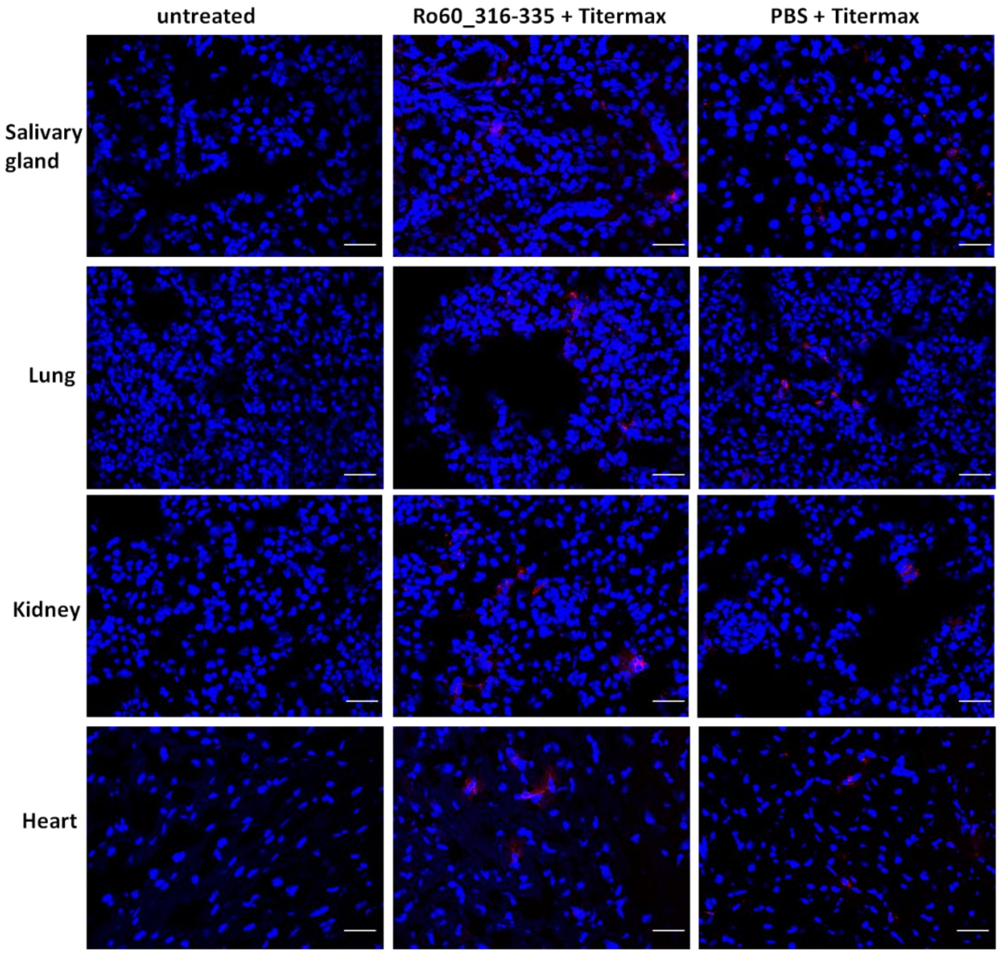

Supplementary Figure 8. Ectopic expression of MHC II molecules in different organs in mice. Mouse organs including salivary gland, lung, kidney and heart were collected from mice treated with Ro60_316-335 peptide or PBS emulsified in Titermax as well as from untreated animals. Ectopic expression of MHC II molecules in lacrimal glands were detected by immunofluorescence staining, and the representative micrographs are shown. Bars, 50μm.

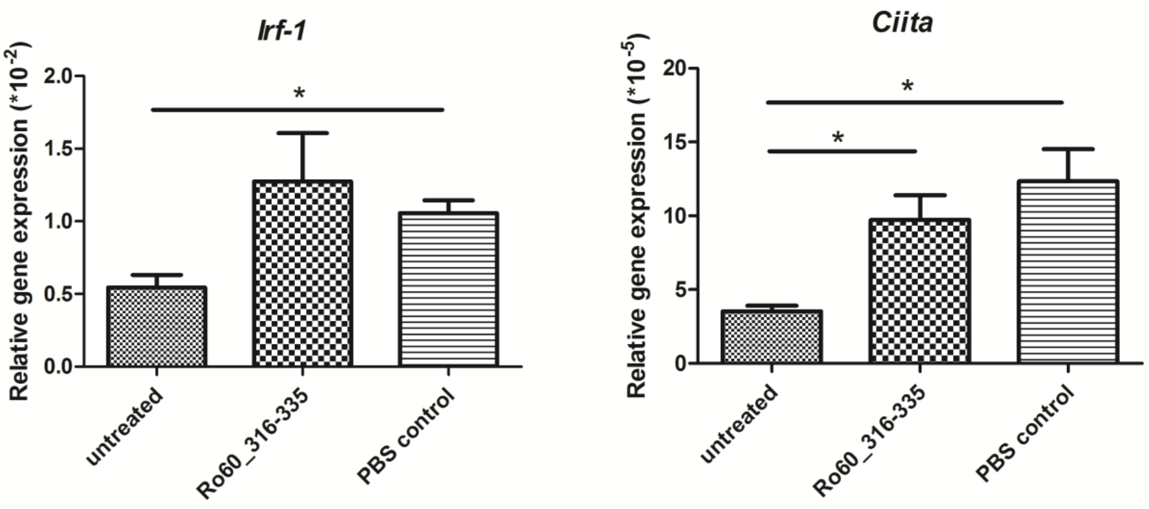

Supplementary Figure 9. Expression of gene *Irf-1* and *Ciita* in murine lacrimal glands. Gene expression of *Irf-1* and *Ciita* in lacrimal glands of non-immunized mice, mice immunized with Ro60_316-335 peptide (n=3) or PBS-treated controls (n=3) emulsified in Titermax. Statistically significant differences between groups were calculated by using Student's test (* p value < 0.05).

Supplementary table 5. Primer lists used for real time qPCR.

1	H2aa-F	TCAGTCGCAGACGGTGTTTAT
2	H2aa-R	GGGGGCTGGAATCTCAGGT
3	H2d1-F	CCTCCTCCGTCCACTGACTCTTA
4	H2d1-R	CTGTCTTCACGCTTTACAATCTCG
5	H2eb1-F	GCGGAGAGTTGAGCCTACG
6	H2eb1-R	CCAGGAGGTTGTGGTGTTCC
7	H2k1-F	AAGGGCGGCTCTCACACTAT
8	H2k1-R	TGCTCCCACTTGTGTTTGGT
9	Irf-1-F	ATGCCAATCACTCGAATGCG
10	Irf-1-R	TTGTATCGGCCTGTGTGAATG
11	Ciita-F	CAGGCTCCCACGGTAGAGA
12	Ciita-R	GGTAGAGATGTAGGGGGTCGG
13	beta-actin-F	AGGTGACAGCATTGCTTCTG
14	beta-actin-R	GCTGCCTCAACACCTCAAC
